# A Broad Set of Different Llama Antibodies Specific for a 16 kDa Heat Shock Protein of *Mycobacterium tuberculosis*

**DOI:** 10.1371/journal.pone.0026754

**Published:** 2011-10-26

**Authors:** Anke K. Trilling, Hans de Ronde, Linda Noteboom, Adèle van Houwelingen, Margriet Roelse, Saurabh K. Srivastava, Willem Haasnoot, Maarten A. Jongsma, Arend Kolk, Han Zuilhof, Jules Beekwilder

**Affiliations:** 1 Plant Research International, Wageningen, The Netherlands; 2 Laboratory of Organic Chemistry, Wageningen University, Wageningen, The Netherlands; 3 Royal Tropical Institute, Amsterdam, The Netherlands; 4 Van 't Hoff Institute for Molecular Sciences, University of Amsterdam, Amsterdam, The Netherlands; 5 RIKILT-Institute of Food Safety, Wageningen, The Netherlands; Statens Serum Institute, Denmark

## Abstract

**Background:**

Recombinant antibodies are powerful tools in engineering of novel diagnostics. Due to the small size and stable nature of llama antibody domains selected antibodies can serve as a detection reagent in multiplexed and sensitive assays for *M. tuberculosis*.

**Methodology/Principal Findings:**

Antibodies for *Mycobacterium tuberculosis* (*M. tb*) recognition were raised in Alpaca, and, by phage display, recombinant variable domains of heavy-chain antibodies (VHH) binding to *M. tuberculosis* antigens were isolated. Two phage display selection strategies were followed: one direct selection using semi-purified protein antigen, and a depletion strategy with lysates, aiming to avoid cross-reaction to other mycobacteria. Both panning methods selected a set of binders with widely differing complementarity determining regions. Selected recombinant VHHs were produced in *E. coli* and shown to bind immobilized lysate in direct Enzymelinked Immunosorbent Assay (ELISA) tests and soluble antigen by surface plasmon resonance (SPR) analysis. All tested VHHs were specific for tuberculosis-causing mycobacteria (*M. tuberculosis*, *M. bovis*) and exclusively recognized an immunodominant 16 kDa heat shock protein (hsp). The highest affinity VHH had a dissociation constant (KD) of 4×10^−10^ M.

**Conclusions/Significance:**

A broad set of different llama antibodies specific for 16 kDa heat shock protein of *M. tuberculosis* is available. This protein is highly stable and abundant in *M. tuberculosis*. The VHH that detect this protein are applied in a robust SPR sensor for identification of tuberculosis-causing mycobacteria.

## Introduction

For centuries, Tuberculosis (TB) has been a severe health problem all over the world. Currently, it is estimated that the microbe *Mycobacterium tuberculosis* causes 9.4 million new cases of TB each year [1]. Recent and alarming developments around TB comprise the appearance of multidrug-resistant strains and co-infection with HIV, both of which reduce the lifetime of tuberculosis patients significantly. Accurate and rapid diagnosis of active TB is essential for both control of disease epidemic, and treatment of infected patients. Current diagnostic methods for TB include DNA-based, biochemical and serological approaches [2]–[6] but none of these methods is yet appropriate for the high-throughput, rapid, reliable and low-cost detection of TB in an affordable near-patient test.

Classical detection methods for infectious diseases such as TB rely on rapid immunological detection. Use of recombinant antibodies may facilitate multiplex design of protein chips, for diagnosing several potential pathogens in parallel [7]. Furthermore, they can be deployed in novel bio-sensing systems such as nanowires with very high sensitivity and potential for near patient testing [8]. Llama antibody fragments (VHHs) are particularly suited for these applications due to their compact size (15 kDa) [9] and remarkable physicochemical stability [10]–[12]. Furthermore, they were shown to display many additional advantages over other recombinant antibodies, regarding cost of production, specificity, affinity, and especially stability under conditions of diagnosis in the field, which would make them suitable as detection units in biosensors [13].

In the present study, our objective was to select and produce VHHs capable of recognizing *Mycobacterium tuberculosis* antigens. VHHs were selected by phage display from a library generated from an immunized alpaca, and characterized. All characterized VHHs bound to the same target – the immunodominant 16 kDa heat shock protein of *M. tuberculosis* - despite having highly diverse sequence profiles. The utility of the selected VHHs in sensor devices were demonstrated using a surface plasmon resonance set-up.

## Results

### Generation of recombinant antibodies

**Figure 1:**
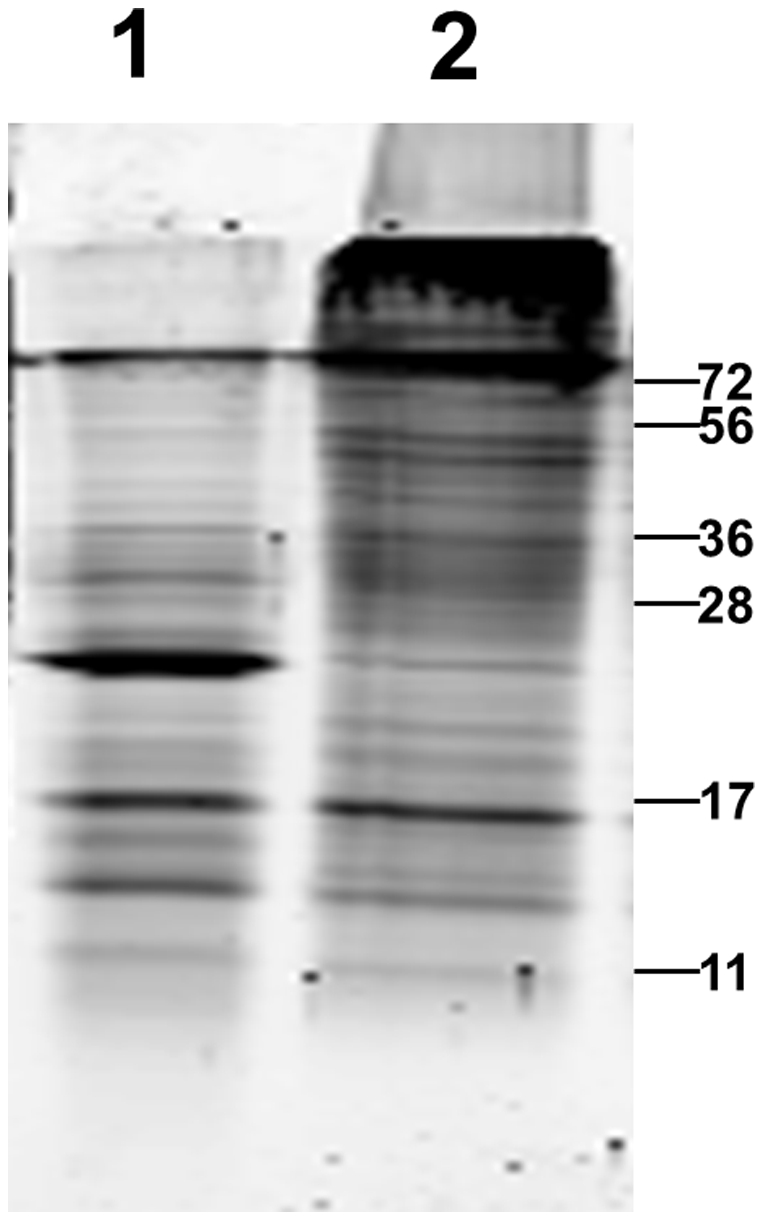
Whole and enriched protein lysate of *M. tuberculosis*. Lysate enriched for 24 kDa, 16 kDa and 70 kDa *M. tuberculosis* protein (lane 1) and whole *M. tuberculosis* lysate (lane 2). Shown is a 15% SDS PAGE gel, stained by SYPRO ruby. Indicated are the positions of relevant size markers in kDa.

Recombinant antibodies for *M. tuberculosis* detection were obtained by phage display. A VHH phage display library with 10**^7^** clones was constructed from the lymphocyte RNA of an alpaca immunized with *M. tuberculosis* lysate. This library was subjected to two different methods of phage display selection: The first method deployed a direct selection on *M. tuberculosis* protein, enriched for 24 kDa, 16 kDa and 70 kDa proteins (Fig. 1). The second method used a depletion strategy, where non-specific phages were removed using total protein of non-tuberculosis mycobacteria, and positive binders were selected on lysate of *M. tuberculosis*. Both selection methods were carried out for three selection rounds. After the last selection, VHH-encoding DNA inserts from both selection methods were mass-excised and transferred into an expression vector.

**Figure 2:**
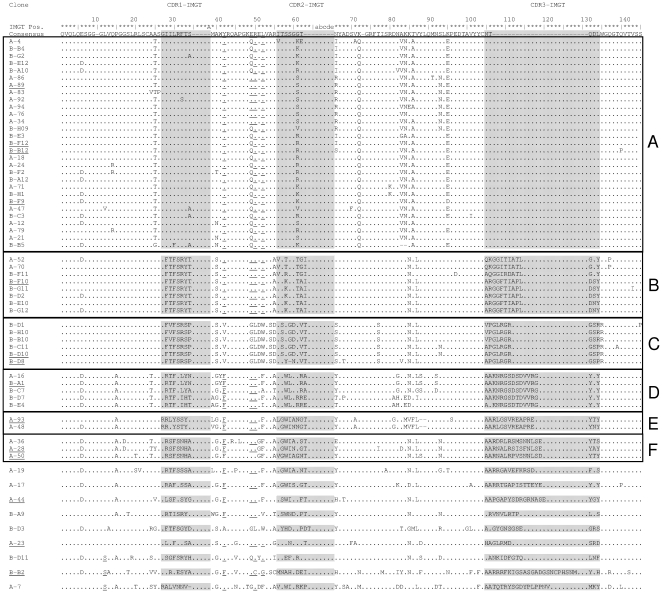
*M. tuberculosis* binding VHH antibody fragments. Protein sequence of 62 selected VHH antibody fragments selected by phage display for binging to *M. tuberculosis*. Dots indicate sequence identity, and dashes indicate gaps. The three complementarity determining regions CDR1, CDR2 and CDR3 are shaded. Characteristic VH-VHH hallmark substitutions (Leu12Ser, Val42Phe or Val42Tyr, Gly49Glu, Leu50Arg or Leu50Cys and Trp52Gly or Trp52Leu (the last substitution is less well conserved) (the ImMunoGeneTics system [52] was followed) [15] are underlined. The 12 clones selected for further investigations are underlined. VHH protein sequences labeled A-x (with x = 1–96) resulted from direct selection using semi-purified protein antigen, protein sequences labeled B-yx (with y = A–F and x = 1–12) were achieved by depletion method.

For each selection method DNA sequences of 96 clones were analyzed. Ninety two of the 192 sequences were rejected because they suffered from premature stop codons or the technical quality of the sequencing reactions was poor. After removal of redundant sequences, 62 unique protein sequences remained, which are shown in Figure 2.

Based on protein similarity, VHH sequences could be clustered into 6 groups (A, B, C, D, E and F) consisting of 29, 8, 6, 5, 2 and 3 members respectively (Fig. 2), while 9 sequences could not be grouped. Both panning methods raised antibodies belonging to the groups A, B and D, suggesting the presence of a dominant antigen either during alpaca immunization or in vitro selection. Grouping was mainly based on the antigen binding regions (CDRs), where most sequence variation occurs. The length of the CDR3 region represents the dominant difference between all single sequences and sequence groups, ranging from five residues in group A to 27 residues in sequence B-B2 (Fig. 2). The variations in protein sequence and length of the antigen binding regions suggests that proteins from different origins with a broad range of sequence diversity were selected.

Sequence groups were inspected for amino-acid substitutions which could classify them as VHHs, and distinguish them from canonical antibodies with heavy and light chain. Most of these substitutions (at positions 42, 49, 50 and 52: underlined in Fig. 2) could be regarded as adaptations to the absence of a light chain [14], [15] resulting in a more soluble VHH fragment. Most prominently, the hydrophobic leucine at position 50 has changed to a water-soluble arginine in 87% of the inspected sequences. Other typical VHH substitutions occur at a lower frequency (15% V42→F; 64% V42→Y; 50% G49→E; 1.6% L50→C; 63% W52→L and 1.6% W52→G).

### Specificity of the selected antibodies

To characterize their properties into more detail, 12 clones (A-23, A-28, A-44, A-50, A-89, A-93, B-A1, B-B12, B-D8, B-D10, B-F9 and B-F10), representing the 6 groups and 3 single sequences, were expressed in *E. coli* at 1 liter scale. Reactivity to *M. tuberculosis* protein - already used for coating in phage display - was assessed in a direct ELISA. All tested VHHs reacted with *M. tuberculosis* protein, but not with *E. coli* protein (not shown), indicating that the selected antibodies could specifically recognize *M. tuberculosis*. The four clones with highest binding capability in ELISA (A-23, A-44, A-50 and B-F10) were used for further analysis.

**Figure 3:**
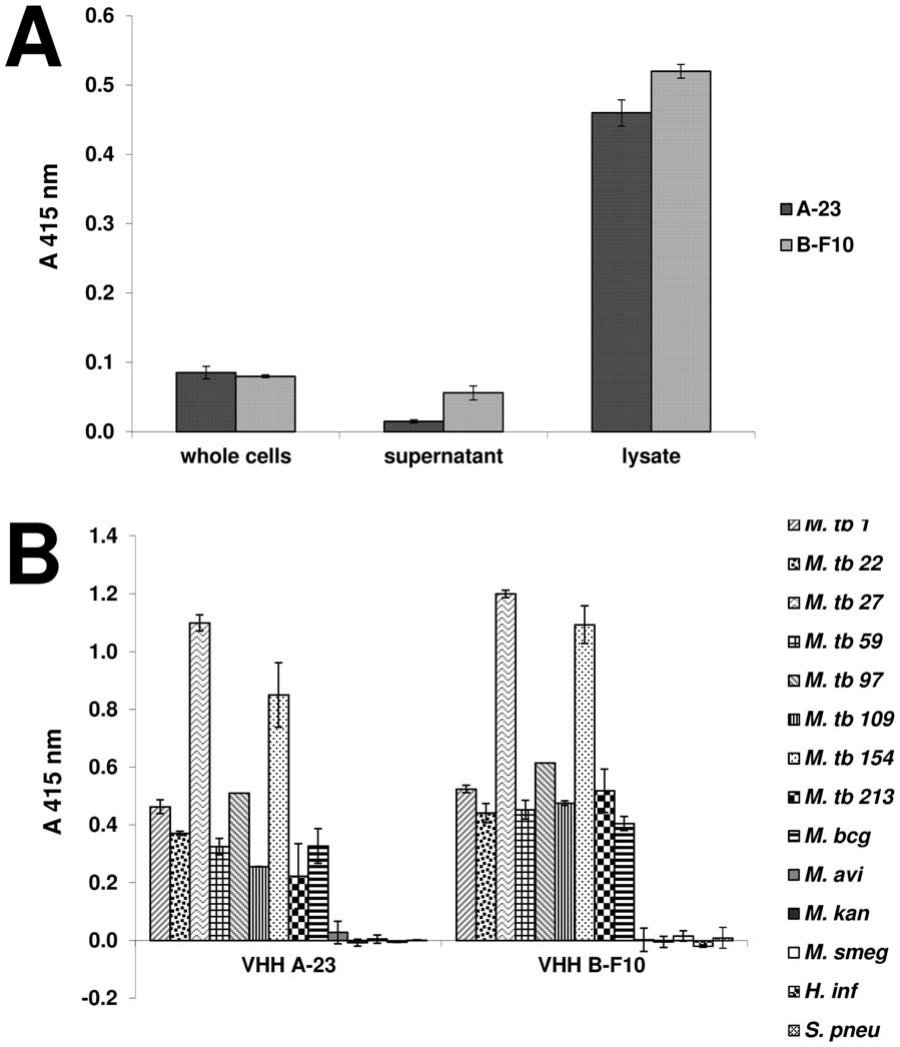
Direct ELISA to confirm specific binging of VHH antibody fragments to tuberculosis lysate. VHH antibody fragments A-23 and B-F10 with A) *M. tuberculosis* whole cells, *M. tuberculosis* cell lysate and media in which *M. tuberculosis* bacteria were grown. B) different *M. tuberculosis* lysates as well as lysates of *M. bcg*, *M. avium*, *M. kansasii*, *M. smegmatis, S. pneumoniae and H. influenzae*. Measurements were performed in duplicate, expressed as means ± SD.

To investigate the capability of the VHH antibody fragments to distinguish *M. tuberculosis* from other mycobacteria, further direct ELISA experiments were carried out. For this purpose different lysates of *Mycobacterium tuberculosis* substrains as well as other *Mycobacterium* species and respiratory pathogens were tested. VHH antibody fragments reacted well with all *M. tuberculosis* substrains and *M. bcg* in different intensities, but not with *M. avium, M. kanssasi*, *M. smegmatis*, *S. pneumoniae* and *H. influenzae* (Fig. 3B). Thus, the selected antibodies displayed selectivity for *M. tuberculosis*.

### Characterization of VHH antibody

The subcellular localization of the antigen recognized by the four selected VHHs (A-23, A-44, A-50 and B-F10) was further investigated. To this end, *M. tuberculosis* whole cells, *M. tuberculosis* cell lysate and the media in which *M. tuberculosis* bacteria had grown was tested in direct ELISA. In all experiments signal was calibrated with those of the media (Middlebrook 7H9) or the buffer (phosphate-buffered saline). It appeared that the VHHs bind most strongly to the *M. tuberculosis* protein lysate (Fig. 3A), indicating that they recognize an intracellular antigen.

**Figure 4:**
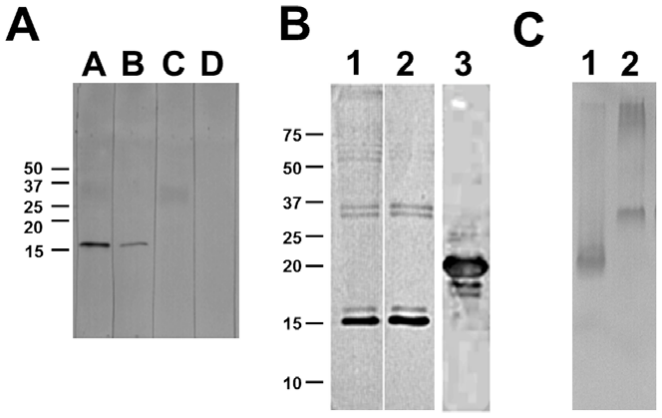
Western blot to discover antigen of VHHs. A) Western blot using VHH A-23 as a probe. 9 µg *M. tb 1* lysate were run on a 15% SDS-PAGE gel in lanes A–D, and transferred to a nitrocellulose membrane. Lane A: incubated with VHH A-23 and detected using anti-VSV-HRP; Lane B: incubated with VHH A-23 and detected using anti-HIS-HRP; Lane C: incubated with detection antibody anti-VSV-HRP; Lane D: incubated with detection antibody anti-HIS-HRP. B) Western blot analysis to confirm the specificity of VHH A-23. Lane 1: 9 µg *M. tb 22* lysate detected by monoclonal mouse 16 kDa antibody, using anti-mouse-HRP secondary antibody; Lane 2: 9 µg *M. tb 22* lysate detected by VHH A-23, using anti-VSV-HRP secondary antibody. Lane 3: 3 µg of purified recombinant 16 kDa protein detected by VHH A-23, using anti-VSV-HRP secondary antibody. Due to the tags added for purification and detection purposes the calculated mass of the recombinant 16 kDa protein is 21 kDa. Indicated are the positions of relevant size markers in kDa. C) Western blot analysis of native PAGE analysis. Lane 1: 5 µg *M. tb 27* lysate; Lane 2: 1 µg of purified recombinant 16 kDa protein. Detection was by VHH A-23, using anti-VSV-HRP secondary antibody.

To identify the target protein of the four selected VHH antibody fragments, Western blot analysis of *M. tuberculosis* lysate was carried out. As exemplified in Figure 4A for VHH A-23, all four tested clones (A-23, A-44, A-50 and B-F10) showed binding to a protein of 16 kDa size. Testing of a number of other selected VHH antibodies (A-28, A-89, A-93, B-A1, B-B12, B-D8, B-D10 and B-F9) revealed that all VHHs bind to a 16 kDa protein (data not shown).

A 16 kDa heat shock protein of *M. tuberculosis* has been reported before to represent an immunodominant protein and its gene was cloned [16]–[20]. To confirm that the band on the western blot corresponded to the 16 kDa hsp reported in the literature, two experiments were performed. Firstly a Western blot analysis was performed, testing the VHH A-23 side-by-side to the mouse monoclonal antibody MoAb F24, which specifically recognizes the 16 kDa hsp [21]. Both tested antibodies bound an identical pattern of bands with a major band around 16 kDa (Fig. 4B, lane 1 and 2). Secondly, the gene encoding the 16 kDa hsp of *M. tu*berculosis was cloned and expressed in E .coli. Purified 16 kDa heat shock protein of E. coli was analyzed by Western blotting. VHH A-23 showed reactivity to the recombinant 16 kDa hsp (Fig. 4B, lane 3). Similar results were obtained for the other VHHs tested (A-28, A-89, A-93, B-A1, B-B12, B-D8, B-D10 and B-F9). Both tests confirm that the 16 kDa hsp from M. tuberculosis is the antigen recognized by these VHHs. When, prior to western blot, antigen preparations were not subjected to denaturing, the reactivity of the antigen was not altered (Fig. 4C).

**Figure 5:**
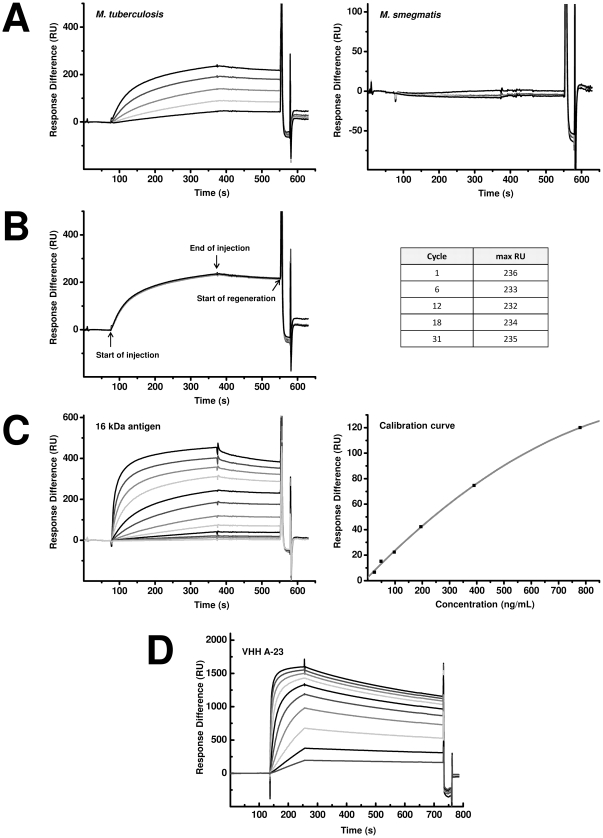
Surface plasmon resonance analysis to show specific binding of VHHs to soluble antigen. A–C) VHH A-23 immobilized at 3818 RU on a CM5 sensor chip in Fc4 and VHH M200 [22] immobilized at 3851 RU in the reference channel Fc3. Sensorgrams were corrected by subtracting the signal from the reference flow channel Fc3. A) Sensorgrams obtained after injection of *M. tuberculosis* (left) and *M. smegmatis* (right) lysate. 50 µL lysate at concentration of 3.8 (lowest), 7.5, 15, 30 and 60 (highest) µg/mL were injected at a constant flow (10 µL/min.). B) Sensorgrams obtained after repeated injection of *M. tuberculosis* lysate. 50 µL lysate at concentration of 60 µg/mL were repeatedly injected at a constant flow (10 µL/min.). Sensor was regenerated with 5 µL of a 10 mM HCl solution at 10 µL/min after each run. C) Left: Sensorgrams achieved after injection of purified recombinant 16 kDa antigen. 50 µL lysate at concentration of 24 (lowest), 49, 98, 185, 391, 781, 1.6×10**^3^**, 3.1×10**^3^**, 6.3×10**^3^**, 12.5×10**^3^**, 25×10**^3^** and 50×10**^3^** (highest) ng/mL were injected at a constant flow (10 µL/min.). Right: Calibration curve of purified recombinant 16 kDa antigen as obtained by BIAevaluation using the 4 parameter fit. D) Sensorgrams achieved after injection of VHH A-23. Purified recombinant 16 kDa antigen was immobilized at 4100 RU on a CM5 sensor chip in Fc2 and inactivated Fc1 was used as reference. 100 µL VHH at concentration of 0.025 (lowest), 0.05, 0.1, 0.2, 0.4, 0.8, 1.6, 3.2, 6.3 and 12.5 (highest) µg/mL were injected at a constant flow (50 µL/min.). Sensorgrams were corrected by subtracting the signal from the reference flow channel Fc1. To obtain the dissociation equilibrium constant (K_D_) the sensorgrams were fitted by a global Langmuir 1∶1 model (BIAevaluation software) using the six lowest VHH concentrations.

The ability of the VHHs to recognize soluble antigen in a biosensor set up was tested in a surface plasmon resonance analysis. VHH A-23 was immobilized at 3818 RU in Fc4 on a CM5 sensor chip. As a reference, VHH M200, selected for recognition of Foot-and-mouth disease (FMD) [22], was used for immobilization at 3851 RU in channel Fc3. Different concentrations of *Mycobacterium* lysates (*M. tb1*, *M. tb27* and *M. smeg*) were run over both flow cells. *M. tb 1* and *M. tb27* bound to VHH A-23 on the CM5 sensor chip, whereas it did not bind to the reference Fc3 (not shown). Lysate of *M. smeg* showed neither binding to VHH A-23 nor to VHH M200. These results are in agreement with the results obtained by ELISA and demonstrate that VHH A-23 is capable of specifically binding the soluble *M. tuberculosis* antigen (Fig. 5A, shown for *M. tb1* and *M. smeg*).

The reproducibility of antigen detection by the A-23 biosensor was investigated using whole *M. tuberculosis* lysate *M. tb1*. The lysate was injected at highest concentration following approximately 8 cycles of antigen binding and regeneration. The maximal RU signals were constant over 31 cycles with a mean of 234±2 suggesting that regeneration conditions (10 mM HCl) do not affect the quality of the immobilized VHH (Fig. 5B). The binding between purified recombinant hsp and a selection of VHH (A-23, A-44, A-50, A-89, B-A1, B-B12, B-D8, B-D10, B-F9 and B-F10) was investigated. Among these, the binding by A-23, A-50, A-44 and B-F10 was found to be the strongest, and binding constants were determined for these VHH. The K_D_ value of B-F10 was found to be best (4×10^−10^ M), while A-23, A-50 and A-44 had slightly lower affinities (2.4×10^−9^ M, 2.2×10^−9^ M and 3.7×10^−9^ M, respectively).

To quantify the antigen present in *M. tuberculsosis* lysates, different concentrations of recombinant 16 kDa antigen (24 ng/mL to 50 µg/mL) were injected for calibration. By plotting the lower concentrations of 16 kDa antigen against the responses in the biosensor, a linear calibration curve was obtained from a level above the limit of detection of 24 ng/mL (Fig. 5C). The signal in the VHH coated flow cell was almost linear up to the concentration of 780 ng/mL and an RU of 119.5. At higher concentrations the response leveled off. For accuracy, only values below 120 RU were used to calculate the % of antigen in the lysate. From the obtained calibration curve, the amount of antigen in the whole protein content of the lysate was determined to be 6.6±0.6% in *M. tb1* and 11.7±0.4% in *M. tb27* whereas *M. smeg* contained 0% of the 16 kDa antigen.

## Discussion

The present study describes the successful selection of recombinant VHHs specific for *Mycobacterium tuberculosis*. A panel of recombinant antibodies was selected, which distinguish a number of *M. tuberculosis* strains from non-tuberculosis mycobacteria. One of the selected VHH molecules was successfully incorporated into an SPR-biosensor to detect and identify *M. tuberculosis*. To our knowledge this is the first description of recombinant llama antibody fragments specific for infectious *M. tuberculosis*.

Two different phage display selection methods were used, one by depletion of the library from non-tuberculosis lysates, and another by using a partially purified protein preparation. The depletion method aimed to select highly specific *M. tuberculosis* VHH antibody fragments against potentially a very wide range of antigens from the lysate, whereas the direct panning method aimed to select VHH antibody fragments specific for the three dominant proteins in the sample. We expected a large sequence variety, and indeed, a broad spectrum of 68 unique sequences was obtained. Out of which six sequences were found to be the same from both selection methods, suggesting that both procedures overlapped in their selectivity for an epitope. Investigations by ELISA with a range of different mycobacteria revealed that the selected llama antibody fragments allowed specific detection of all tested tuberculosis-causing species, and distinguish them from non-infectious mycobacteria such as *M. kansasii*, *M. avium* and *M. smegmatis* and other respiratory pathogens such as *S. pneumoniae, and H. influenzae* (Fig. 3B). Apparently, both selection methods lead to antibodies that specifically recognize the *Mycobacterium tuberculosis* complex.

Earlier, selection of several mouse single-chain fragments (scFvs) reactive with *M. tuberculosis* culture proteins by phage display was reported [23], and very recently also chicken scFv antibodies for 16 kDa hsp [24]. In the present work, VHH was preferred over scFv, because scFvs are larger, and often suffer from poor stability, unless genetically engineered [25], [26], while VHHs often display high stability over long periods of exposure to ambient temperature [10], which allowed for extensive re-use of the SPR chip.

Surprisingly, Western blot analysis showed that all 12 tested VHHs from different clusters bind to the species-specific 16 kDa hsp (Fig. 4B), despite the high sequence diversity among the clusters (Fig. 2). Apparently also in alpaca the 16 kDa hsp behaves as an immunodominant antigen, and possibly there are several different epitopes. The specificity for the 16 kDa hsp is consistent with studies into the conservation of this protein among mycobacteria. Southern blot analysis of genomic DNA of several mycobacteria, using the coding sequence of the 16 kDa hsp gene as a probe [27], showed the absence of a homologue of the 16 kDa hsp in non-tuberculosis species. Only the tuberculosis related *M. bovis* bacillus Calmette-Guérin (BCG) strain showed a specific hybridization, which is in agreement with our observation that 16 kDa hsp specific VHHs (A-23, A-44, A-50 and B-F10) showed binding to *M. tuberculosis* and *M. bovis* BCG but not to any other tested *Mycobacterium* species in ELISA (Fig. 3B). Similar results were observed by Coates *et al* for the mouse monoclonal antibody [28] TB68, which binds to the 16 kDa hsp of all tested strains of *M. tuberculosis* and *M. bovis* species [29].

The 16 kDa hsp is known under several names (Hsp16.3, sHsp16 and Acr) [30] and shares a low sequence homology with the α-crystallin-related small heat shock protein family [31]. It has been suggested that this antigen acts *in vitro* as an adenosine triphosphate-independent chaperone and may occur as a dodecamer, or as monomers [17], [32]. The form of antigen that is recognized by the VHH is probably the monomeric structure. The hsp was isolated from bacteria by a denaturing treatment, which would cause monomer formation, and reactivity of the antigen is not very different after western blotting from a native gel or a denaturing gel (Fig. 4). Also heat denaturing of antigen hsp did not change its mobility in native gels (not shown). Thus, probably the antigen is recognized in monomeric form, while we have no dodecameric form available for testing its reactivity.

Several murine monoclonal antibodies against the prominent hsp antigen of *M. tuberculosis* have been selected in the past [21], [29], [33]. It has been subcloned, overexpressed and purified [17], [20]. The results of the SPR experiments comparing the *Mycobacterium* lysates to purified recombinant 16 kDa hsp show that 7 to 12% of cellular protein consists of 16 kDa hsp. This would correspond to ±6 million copies of 16 kDa hsp per cell, taking into account its molecular weight, and an estimated 160 fg total protein per cell [34]. The concentration of hsp in the total protein content of *M. tb* is comparable with Lipoarabinomannan (LAM), the major glycolipid of the outer cell wall which may represent up to 15% of the total bacterial weight [35] and is used for tuberculosis detection in urine samples [36]. Due to its rich presence in bacteria lysate, the highly expressed 16 kDa hsp seems to be a relevant biological target for TB diagnostic assays [34]. Nevertheless it may be worthwhile to develop stable and high affinity VHH antibodies to secreted *M. tb* proteins such as the 24 kDa protein [37] for diagnosis.

The 16 kDa hsp is a cytosolic protein, as hardly any ELISA signal of the VHHs to whole cells or culture medium of the *M. tuberculosis* cultures could be observed, while a strong signal occurred in the cell lysate (Fig. 3A). These observations are in agreement to reports of others, who report that the 16 kDa hsp is not released into the culture supernatant [38], [39], but is peripherally associated with the membrane [40]. One consequence of this subcellular localization is that lysis of bacteria will be necessary before detection in sputum. One might argue that a secreted antigen like the 24 kDa protein [37], would be more convenient for immunological detection. On the other hand, most tuberculosis-detection methods based on nucleic-acid amplification also require vigorous disruption of sputum and mycobacterial cells and a secreted antigen may be too dilute in sputum [41].

Most serological tests for tuberculosis screen for reactivity in the patient's serum to specific *M. tuberculosis* antigens, such as the 16 kDa hsp [20], [42], [43]. However, such serum-reactivity tests have limited value, as antibodies remain present after BCG vaccination, after successful treatment of the infection and during latent infection, which complicates the diagnosis of active TB disease. Therefore, more efforts have been directed towards direct detection of *M. tuberculosis* antigens in sputum, serum, urine and cerebrospinal fluid of patients, and there VHH antibodies against the 16 kDa hsp may represent a useful tool for the development of diagnostic tests.

An SPR biosensor using one of the selected VHHs allowed detection of recombinantly produced 16 kDa hsp antigen of *M. tuberculosis*. Moreover, the SPR sensor was able to positively identify crude lysates of *M. tuberculosis*, enabling the specific detection of tuberculosis-causing infectious agent. The sensor system proved to provide reproducible results after a significant number of regenerations, which is an important attribute for a sensing device. Importantly, further experiments should prove the value of an SPR sensor in the identification of *M. tuberculosis* in patients' material. At its current sensitivity (24 ng of 16 kDa hsp per mL; Fig. 5C), the limit of detection would be about 10^6^ cells per mL, when taking into account that 16 kDa makes up approximately 10% of total *Mycobacterium* protein and a cellular protein content of 150 fg/cell [34]. The current limit of detection using microscopy is around 10,000 cells per mL.

Sensitivity of the SPR system could be greatly enhanced by immobilization of VHHs in an oriented way, leading to increased exposure of antigen-binding sites. Such orientation of antibodies like VHH could be achieved by site-specific biotinylation of the VHH, or other, covalent immobilization methods [44]. SPR results indicated a dissociation constant of 4×10^−10^ M for B-F10. Whereas other VHH with 10-fold higher affinity have also been reported [45] [45], affinity maturation of the VHH by rounds of mutagenesis and selection could significantly improve the affinity of the antigen, and thereby the sensitivity of the sensor [46] and finally multivalent and multispecific VHHs could be engineered for strongly enhanced affinity for the antigen. VHHs against a broad range of antigen targets could be selected by tuning the phage display selection method and the specificity and sensitivity of *M. tb* detection could be greatly enhanced by the combination of VHHs for rapid, near patient multiplexing in the near future.

SPR is one of the more established biosensor principles. Other methodologies, such as piezoelectronic immunosensors or fluorescent nanoparticles coupled with flow cytometry, and nanowires are further away from application in practice [8], [47], [48]. Still, SPR equipment is hardly compatible with field settings in countries where tuberculosis diagnosis is needed most. Recent efforts to implement portable SPR devices in tuberculosis diagnostics [49] may contribute to the solution of this problem.

## Materials and Methods

### Bacteria samples

All mycobacteria (Royal Tropical Institute, Amsterdam, The Netherlands) were grown in Middlebrook 7H9 medium (Difco, BD, Sparks, MD, USA) supplemented with 10% OADC (BBL, BD) until mid-log phase and heat-killed at 80°C for 20 min. Other respiratory pathogens (*Streptococcus pneumoniae D39* and nontypable *Haemophilus influenzae R2866* [Radboud University Nijmegen Medical Center CUKZ, Nijmegen, The Netherlands]) were grown on chocolate agar. Heat-inactivated bacteria were used as ‘whole cells’ to test binding of antibodies to surface proteins. To obtain the lysate of *Mycobacterium* as antigen source, bacteria were centrifuged at 13,000 g for 5 min after heat killing. After 2 washing steps with phosphate-buffered saline (PBS) to remove all media the bacteria pellet was resuspended in PBS. 500 µL of bacterial suspension was lysed with 0.6 g zirkonia/silica 0.1 mm (BioSpec Products Inc, Bartlesville, OK, USA) in a Retch MM 301 (Retch GmbH, Germany) for 15 min at 30 hertz. To remove soluble particles, the lysate was centrifuged for 5 min at 13,000 g. The obtained supernatant was used as cell lysate. Protein concentration of mycobacterium samples were determined with the Pierce 660 nm Protein assay using Nanodrop and albumin as reference protein for the standard curve.

To obtain enriched lysate 2 mL of centrifuged *M. tb* lysate was brought into 20 mM ethanolamine (pH 9.0) and loaded on a 1 mL HiTrap MonoQ column (Pharmacia). After washing extensively with 20 mM ethanolamine (pH 9.0), a linear gradient from 0 to 1 M NaCl in 20 mM ethanolamine (pH 9.0) was applied, and 1 mL fractions were collected. Around 0.4 mM NaCl, a fraction eluted in which proteins of 16 kDa, 24 kDa and 63 kDa were overrepresented, and this fraction was used for subsequent selections.

### Library construction

A VHH library was formed from lymphocyte RNA from a 3-year old female alpaca (GDL, Utrecht University, The Netherlands) as previously described [10]. Experiments with alpacas were approved by the Dutch Animal Ethical Commission (Dier Experimenten Commissie, DEC; permit 06/284) of the Utrecht University, following the Dutch Law on animal experiments (“Wet op de Dierproeven” : http://wetten.overheid.nl/BWBR0003081/geldigheidsdatum_05-09-2011). 100 µg of *M. tuberculosis* lysate was used for the immunization. VHHs were amplified from first-strand cDNA with VHH specific primers lam07, lam08 and lam17 [50]. Amplified fragments were pooled and ligated into the pHEN2 phagemid vector [51] in frame with the M13 gene 3 for expression of VHH-g3p fusion protein. Electroporation of recombinant plasmid into competent *Escherichia coli* TG1 cells resulted in about 10**^7^** individual recombinants. Phage particles were produced in *E. coli* as described earlier [10].

### Selection

The library containing approximately 10^7^ individual clones was panned separately using two different panning strategies.

### Direct Panning Method

The first selection was carried out by panning of the VHH-displayed phage library directly against the *M. tuberculosis* lysate, enriched for 24 kDa protein, 16 kDa protein and another high-molecular weight protein around 70 kDa (Fig. 1). Wells of microtiter ELISA plates (GreinerBioOne, The Netherlands) were coated with 100 µL lysate (10 µg mL**^−1^** in 0.1 M Sodium Carbonate Solution pH 9.6) overnight at 4°C. Plates were emptied the next day and washed 1 time with 200 µL PBS and unspecific binding sites were blocked for 1 h with 250 µL PBS containing 2% (w/v) nonfat powdered milk (2% PBSM). 100 µL phages (10**^11^** cfu mL**^−1^**) were blocked for 30 min with 50 µL PBS containing 10% nonfat powdered milk (w/v) (10% PBSM) before 100 µL of this mix was applied to the wells and incubated for 1 h on a shaker. Excess phages were washed away with 5 washes of 200 µL 0.05% Tween-20 in PBS (0.05% PBST) and 5 washes of 200 µL PBS. Phages from the *Mycobacterium* selection were eluted with 100 µL of 0.1% trypsin in PBS for 15 min. Eluted phages were then used to infect *E. coli* TG-1 cells. The phage populations were amplified and rescued by VCSM13 helper phage to generate phage displaying VHH to be used for the next round of panning. To raise the selection stringency, the number of washing steps before trypsin elution was increased by 5 after each panning round. After three rounds of panning, the in- and output were titrated to monitor the success of the selection.

### Depletion Panning Method

Targets were immobilized overnight at 4°C in microtiter ELISA plate wells (GreinerBioOne, The Netherlands) using the following conditions: 6 wells were coated with 100 µL *Mycobacterium* mix (lysate of *M. kansasii*, *M. avium*, *M. fortuitum* and *M. chelonae*, 3 µg mL**^−1^**) in PBS and one well with 100 µL lysate of *M. tuberculosis* (3 µg mL**^−1^**) in PBS. The next day, plates were emptied and washed one time with 200 µL PBS and blocked for 1 h with 250 µL 2% PBSM. 300 µL phages (10**^11^** cfu mL**^−1^**) were blocked for 30 min with 100 µL 10% PBSM before 100 µL of this mix were applied to the emptied lysate-containing (*Mycobacterium* mix) well and incubated for 15 min on a microtiter plate shaker. The supernatant was then transferred to the next lysate-containing (*Mycobacterium* mix) well and incubated for 15 min under shaking. This procedure was repeated for the remaining 4 wells coated with the *Mycobacterium* mix lysate. Finally the supernatant was transferred to the well coated with *M. tuberculosis* lysate and incubated for 30 min on the shaker. Excess phages were washed away with 5 washes of 200 µL 0.05% PBST and 5 washes of 200 µL PBS. Phages from the *Mycobacterium* selection were eluted with 100 µL of 0.1% trypsin in PBS for 15 min. Eluted phages were then amplified, rescued and re-used. In additional panning rounds stringency was increased by increasing the number of washing steps by 5 after each panning round.

### Recloning of selected VHHs for expression

For both panning methods, plasmids from selected phage pools were extracted using QIAprep Spin Miniprep Kit (250) (QIAGEN, The Netherlands). DNA was cut using the unique *Pst*I and *Not*I restriction sites and then purified using the Jetquick gel extraction kit (Genomed, Germany). VHH sequences were then bulk-ligated into a *Pst*I and *Not*I digested PRI-VSV expression vector, a strong expression vector for expression in the periplasm, based on the backbone of the pRSET-A vector (Invitrogen, The Netherlands). Expression of the recombinant VHH was under control of the T7 promoter. The C-terminus of the VHH was fused to a VSV-tag (YTDIEMNRLGK) for detection purposes along with a 6× His tag for purification purposes followed by a stop codon. For cloning purposes a *NotI* restriction site was introduced between the VHH and the VSV-tag.

Constructs were introduced into E. coli XL-1 blue. 96 randomly picked colonies were used for colony PCR with the primer T7 (5′- TAATACGACTCACTATAGG -3′) and AS2 (5′- GCTAGTTATTGCTCAGCGG -3′). Sequencing of 96 colonies from the first panning method resulted in 34 unique VHH protein sequences (labeled A-x, with x = 1–96), for the depletion method in 34 unique full length protein sequences (labeled B-yx, with y = A-F and x = 1–12). All duplicate sequences were omitted. The remaining 62 non-redundant VHH sequences were aligned according to ImMunoGeneTics system [52] for immunoglobulins and classified into different groups. As VHHs had unusual long CDRs, additional gaps were introduced in the numbering at the end of CDR1 and CDR2 and labeled with letters A and a to e, respectively.

Sequences for 12 representatives which were used for further analysis have been submitted to Genbank under accession numbers JN234011–JN234022.

### Expression and Purification of recombinant VHHs

Representative sequences from each panning method were selected from different groups for expression in *E. coli* BL21-AI, a strain which carries a chromosomal insertion of the T7 RNA polymerase gene in the *ara*B locus of the *ara*BAD operon, allowing the expression of recombinant VHH in the presence of L-arabinose. Bacteria were induced to express the VHH and urea-lysed as described previously [10] using PBS as buffer. VHHs were purified using Ni-NTA Superflow resin (QIAGEN, Germany) as reported before [10]. Eluates were dialyzed against 8 liter of PBS in two steps after Ni-NTA metal-affinity chromatography and samples were stored in 1.5 mL batches containing a final concentration of 15% glycerol at −20°C. Protein concentration was determined using Bradford test [53] while successful expression and purification was verified on a 15% sodium dodecylsulphate polyacrylamide gel electrophoresis (SDS-PAGE) gel. As previously shown by Beekwilder et al. [10] the expressed VHHs were the dominant proteins in the *E. coli* cell pellets (data not shown).

### Direct ELISA

Wells of flat-bottom ELISA plates (medium-binding, GreinerBioOne, The Netherlands) were coated with 2 µg mL**^−1^** lysate of *Mycobacterium* species or other respiratory pathogens (*S. pneumoniae and H. influenza*) in PBS and incubated at 4°C overnight. Antigen-coated wells were emptied and washed one time with 200 µL PBS and then blocked with 150 µL PBS containing 4% nonfat powdered milk (w/v) (4% PBSM) for 1 h at room temperature. Wells were emptied and 100 µL VHH (0.5 mg mL**^−1^**) in 2% PBSM was added and binding was allowed to occur for 1 h. Excess VHHs were removed by washing three times with 200 µL PBST, and anti-VSV-HRP conjugate (Sigma Aldrich, Missouri, USA) was added at a 1∶2000 dilution for 1 h in 2% PBSM. Excess conjugate was washed off three times with 200 µL PBST and three times with 200 µL PBS. Subsequently HRP activity was determined by adding 1-StepTM Ultra TMB-ELISA substrate (Pierce, Rockford, IL). The reaction was allowed to proceed in the dark for 10 min and then stopped with 1 M sulphuric acid and the OD was measured at 415 nm in a microtiter plate-reader (TECAN SpectraFluor Microplate Reader).

Similar method was employed for direct ELISA of whole cells and supernatant by using 100 µL for coating.

### Western Blot analysis

Cell lysates containing approximately 9 µg total protein were first boiled and reduced in buffer and electrophoresed on a 15% SDS-PAGE gel and SYPRO ruby (Bio-Rad, Hercules, CA) stained to ensure the full complement of proteins was resolved. For Western blotting, proteins were transferred from an unstained gel to nitrocellulose membranes (Trans-Blot, Bio-Rad, Hercules, CA). Membranes were blocked overnight at room temperature in 4% PBSM on a shaker. The membrane was then incubated with purified VHH (0.1 mg mL**^−1^** in 2% PBSM) for 1 h at room temperature on a shaker. Membranes were washed once in 2% PBSM and three times in 0.1% PBST. Subsequently, membranes were incubated with HRP-conjugated anti-VSV-tag antibodies (1∶2000 in 2% (w/v) PBSM, Sigma Aldrich, Missouri, USA) or anti-HIS-tag (1∶5000 in 2% (w/v) PBSM, Roche, Mannheim, Germany) for 1 h at room temperature on a shaker. Membranes were washed once with 2% PBSM followed by two washing steps with TBS containing 0.1% Tween 20 and two washing steps with TBS. Binding was detected with 3,3,5,5′-Tetramethylbenzidine (TMB) liquid substrate system for membranes (Sigma-Aldrich, The Netherlands). Molecular weight standard was Precision Plus Kaleidoscope (BioRad, Hercules, CA).

### Recombinant 16 kDa protein

The 16 kDa protein was PCR-amplified from *M. tuberculosis* lysate using the primer HSP16.3-PstI-FW (5′- AAAAAAACTGCAGAAAATGGCCACCACCCTT CCC -3′) and HSP16.3-NotI-RV (5′- AAAAAAAAGCGGCCGCGTTGG TGGACCGGATCTGAA -3′) (*PstI* and *NotI* restriction sites are underlined). The digested fragment was inserted in a PstI-NotI cut PRI-AVI expression vector. This vector is similar to the earlier described PRI-VSV expression vector, but the C-terminus of the protein is fused to an AVI-tag™ (Avidity, LLC, Denver, Colorado) and a 6× His tag for purification purposes followed by a stop codon. The construct was transformed into *E. coli* XL-1 blue for multiplication. The cloned hsp sequence is identical to GenBank accession number S79751. Isolated plasmid DNA was transformed into *E. coli* strain BL21-AI for expression. Expression and Ni-NTA metal-affinity chromatography purification was performed as described for VHH. The calculated mass of the recombinant 16 kDa protein was determined to be 21 kDa.

### Surface Plasmon Resonance (SPR)

The Biacore 3000, carboxymethyl dextran sensor chips (CM5), HBS-EP buffer (pH 7.4, consisting of 10 mM 4-(2-hydroxyethyl)piperazine-1-ethanesulfonic acid, 150 mM sodium chloride, 3 mM ethylenediaminetetraacetic acid, 0.005% v/v surfactant polysorbate 20), the amine coupling kit (containing 0.1 M N-hydroxysuccinimide (NHS), 0.4 M 1-ethyl-3-(3- dimethylaminopropyl)carbodiimide hydrochloride (EDC) and 1 M ethanolamine hydrochloride (pH 8.5)) were purchased from GE Healthcare (Uppsala, Sweden).

Selected VHHs were immobilized onto a CM5 surface by the use of the amine coupling kit and the Surface Preparation Wizard as present in the Biacore 3000 control software. The biosensor surface was activated by injecting (35 µL at a flow rate of 5 µL/min) a mixture of EDC and NHS (1∶1; v/v) into one of the four flow channels (Fcs). Then VHH (50 µg/mL diluted in coupling buffer (10 mM sodium acetate, pH 4.0)) was injected and bound to the activated carboxymethylated dextran surface via its primary amine groups, aiming for an immobilization level of 5000 response units (RU). After coupling, the remaining active groups were blocked with ethanolamine hydrochloride (1 M).

In the experimental set-up, a reference (non-tuberculosis binding, M200 [22]) VHH was immobilized in the reference Fc3 and the tuberculosis specific VHH was immobilized in Fc4, both with a final response of approximately 3800 RU. The Biacore 3000 operated at a constant temperature of 25°C and a constant flow of 10 µL/min. 50 µL *Mycobacterium* lysates in HBS-EP buffer, each of different concentration, were injected over the two serially connected Fcs. Regeneration of sensor surface was done with 5 µL of a 10 mM hydrogen chloride (HCl) solution at 10 µL/min after each run. For quantitative analysis, a calibration graph was prepared by using different concentrations of purified 16 kDa antigen in HBS-EP. All sensorgrams were referenced by subtracting the signal from the reference flow channel (Fc3) from Fc4 and were evaluated using the BIAevaluation software.

To check the affinity of different representative VHHs (A-23, A-44, A-50, A-89, B-A1, B-B12, B-D8, B-D10, B-F9 and B-F10) against the 16 kDa hsp, the antigen was immobilized at 4100 RU on a CM5 sensorchip in Fc2 using the amine coupling method. A second flow cell (Fc1) was treated with the same chemical procedure without antigen and used as a reference. At a constant flow rate of 50 µL/min different concentrations of 100 µL VHH ranging from 12.5 to 0.025 µg/mL in HBS-EP were injected 120 s over the two flow cells and followed by a dissociation step of 400 s. The sensor surface was regenerated with 25 µL of a 20 mM HCl solution. The resulting sensorgrams were referenced by subtracting the signal from the reference flow channel (Fc1) from Fc2. To obtain the dissociation equilibrium constant (K_D_) the sensorgrams were fitted by a global Langmuir 1∶1 model (BIAevaluation software) using the six lowest VHH concentrations.
